# Supplementary material for: SARS-CoV-2 nsp14 Exoribonuclease Removes the Natural Antiviral 3′-Deoxy-3′,4′-didehydro-cytidine Nucleotide from RNA
Source: Viruses. 2022 Aug 16;14(8):1790. doi: 10.3390/v14081790 (PMC9415739; doi:10.3390/v14081790)
Supplement: Supplementary file 1 [file viruses-14-01790-s001.zip › ddhCTP_ExoN_Figure_S1.pdf]

Figure S1. Purity of ddhCTP

ddhCTP was synthesized and purified as previously described (*J. Med. Chem.* **2021**, *64*, 15429-15439). The following spectral data are provided to demonstrate purity:

A. ^{31}P NMR of the purified ddhCTP sample in D_2O.

B. Low resolution mass spectrum of the purified ddhCTP sample (ESI$^+$, M+H$^+$, $C_9H_{15}N_3O_{13}P_3$; calculated: 465.98, found 466.0).

C. Analytical HPLC trace of purified ddhCTP sample at 260 nm. Purity: 96%.

A

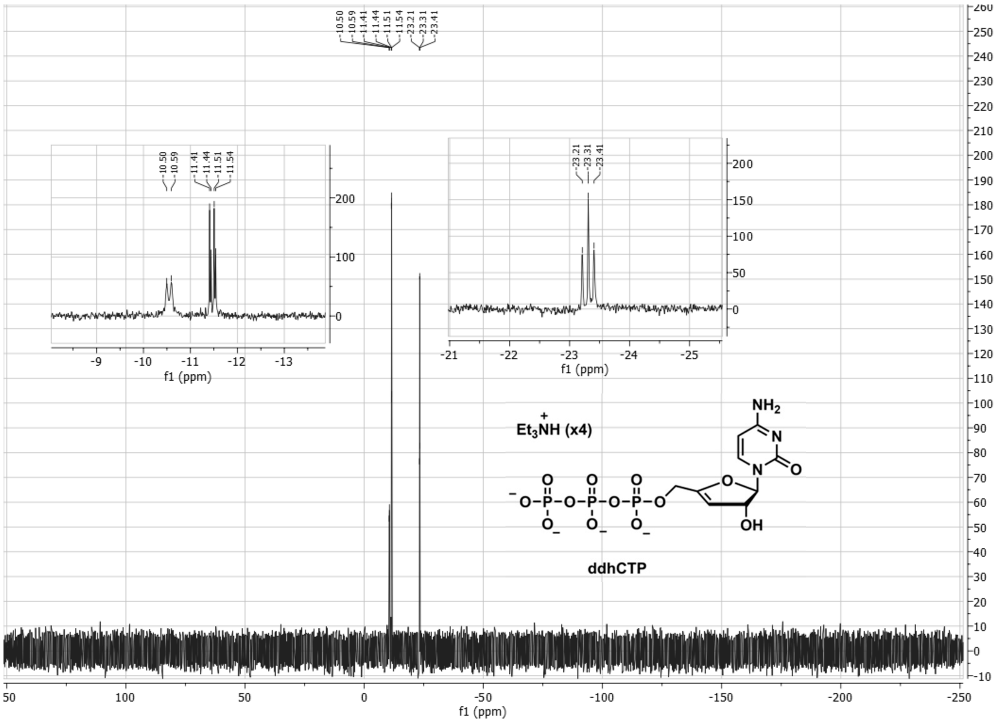

B

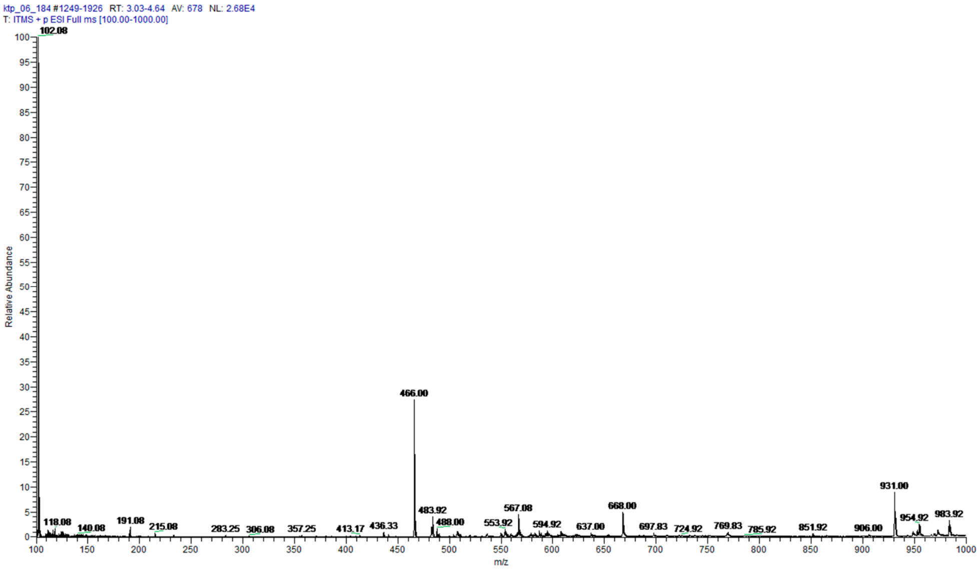

C

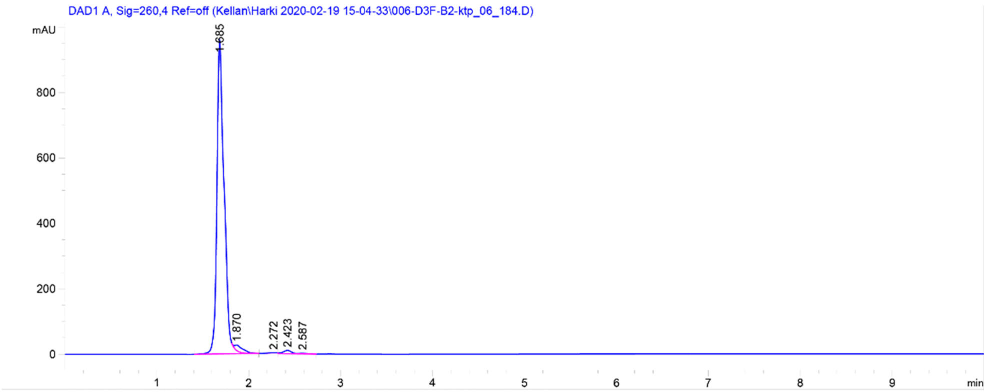

DAD1 A, Sig=260,4 Ref=off (Kellan\Harki 2020-02-19 15-04-33\006-D3F-B2-ktp_06_184.D)
mAU
800
600
400
200
0
1.685
1.870
2.272
2.423
2.587
1
2
3
4
5
6
7
8
9
min
