# Supplementary material for: SARS-CoV-2 nsp14 Exoribonuclease Removes the Natural Antiviral 3′-Deoxy-3′,4′-didehydro-cytidine Nucleotide from RNA
Source: Viruses. 2022 Aug 16;14(8):1790. doi: 10.3390/v14081790 (PMC9415739; doi:10.3390/v14081790)

Figure S2. Representative spectrograms showing band intensities for the gel result in Figure 3b and 3c

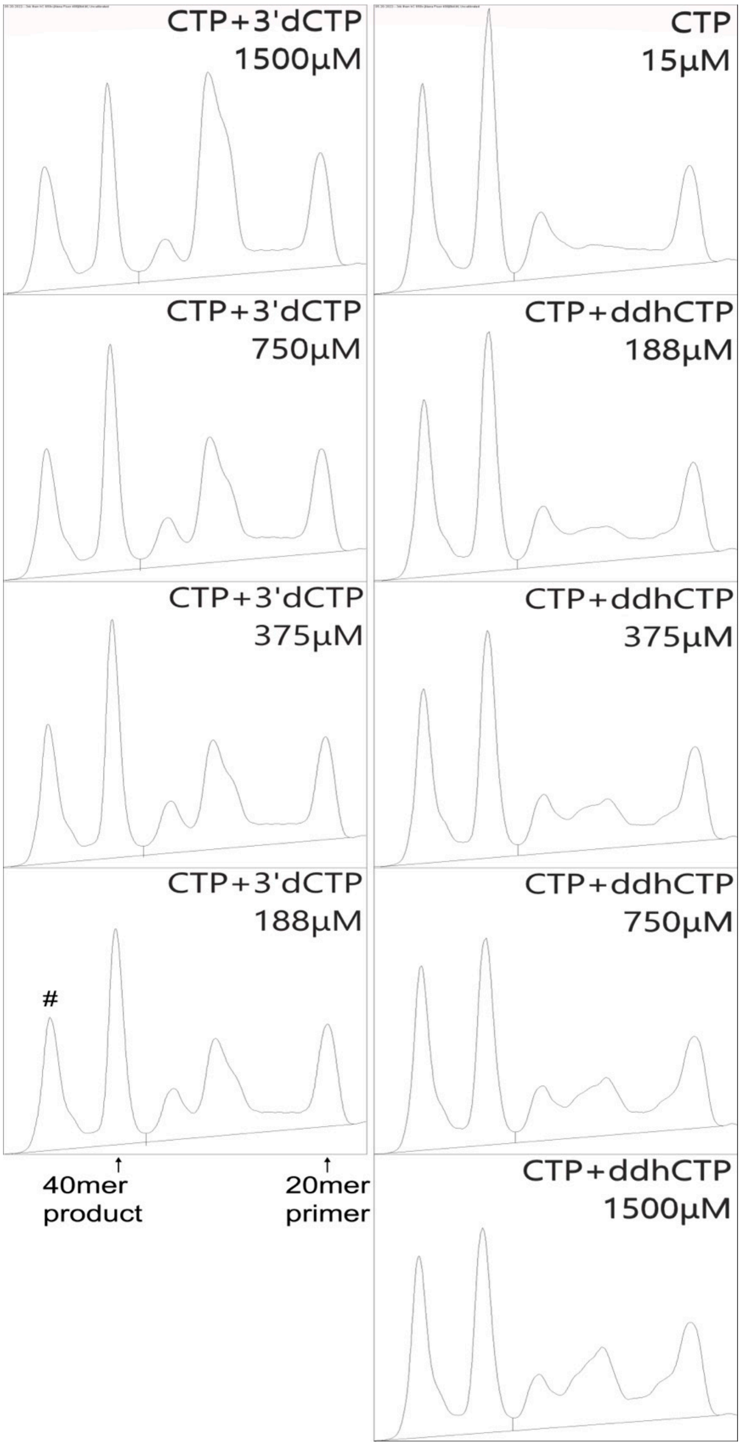

Supplement: Supplementary file 1 [file viruses-14-01790-s001.zip › ddhCTP_ExoN_Figure_S2.pdf]
